# Cultivated Vegetation Shapes Diversity and Stability of Spontaneous Herbaceous Communities in Residential Green Spaces

**DOI:** 10.1002/ece3.73761

**Published:** 2026-05-30

**Authors:** Jia Peng, Jing Liu, Zhihuan Huang

**Affiliations:** ^1^ College of Architecture and Design University of South China Hengyang China

**Keywords:** community stability, cultivated vegetation, residential green spaces, spontaneous herbaceous plants, urban biodiversity

## Abstract

Urban residential green spaces contribute to urban biodiversity by supporting both cultivated and spontaneous herbaceous plants, yet the effects of cultivated vegetation on spontaneous communities remain insufficiently quantified. Here, we investigated the composition, diversity, and stability of spontaneous herbaceous communities across 21 residential green spaces in Hengyang, China. A total of 145 spontaneous species were recorded, including 99 native and 46 non‐native species. Community composition was strongly dominated by occasional species (119 spp.), far exceeding dominant and common species. Cultivated vegetation significantly influenced community diversity, with effects varying among vegetation types. Higher diversity was observed in plots without cultivated vegetation and those dominated by *Cynodon dactylon* , whereas plots dominated by *Ophiopogon japonicus* and mixed vegetation exhibited reduced diversity. Seasonal variation in diversity also depended on vegetation type, with significant spring‐autumn differences detected only in less intensively managed plots. Community dissimilarity and stability both varied across vegetation types and were more closely associated with species frequency structure than with total diversity. Dissimilarity decreased with increasing diversity of dominant and common species, whereas community stability was significantly related to the diversity of occasional species. These findings indicate that cultivated vegetation shapes spontaneous plant communities by altering species frequency structure and habitat conditions, with implications for biodiversity‐oriented design and management of residential green spaces.

## Graphical abstract

Cultivated vegetation shapes the diversity and stability of spontaneous herbaceous communities in residential green spaces, with effects varying across vegetation types. Spontaneous communities were dominated by occasional species, and community stability and dissimilarity were more closely associated with species frequency structure than with overall species richness.

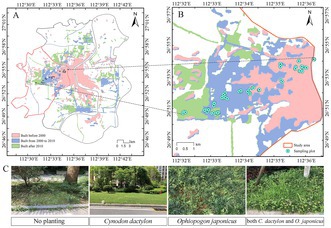

## 1 Introduction

Spontaneous herbaceous plants are non‐cultivated species that colonize, establish, and persist in urban environments through natural regeneration from soil seed banks and seed rain (Del Tredici 2010; Cervelli et al. 2013; Li et al. 2026). Collectively, these species form spontaneous plant communities that reflect the combined effects of environmental filtering and anthropogenic disturbance. Their rapid adaptation to urban conditions, including limited resources and frequent disturbance, makes them a key component of urban ecosystems (Bonthoux et al. 2019; Qiu et al. 2021). In a large‐scale survey of urban vegetation in Beijing, spontaneous herbaceous species accounted for 38.9% of all recorded plant species (Zhao et al. 2010). These species contribute to a range of ecosystem services, including supporting biodiversity, improving soil quality, and regulating microclimate, while requiring substantially lower maintenance than intensively managed landscapes (Ignatieva and Hedblom 2018; Gaw and Richards 2021; Li, Liang, et al. 2023; Li et al. 2026). In addition, some species are integral to traditional diets, cultural practices, and folklore, thereby contributing to human health and well‐being (Gurib‐Fakim 2006; Robinson and Lundholm 2012; Huang et al. 2026). They also play an important role in shaping low‐maintenance, naturalistic urban landscapes with esthetic and recreational value (Yu 2007; Ignatieva and Hedblom 2018).

Despite these benefits, spontaneous herbaceous plants are often regarded as “weeds” and are routinely removed in urban management (Bonthoux et al. 2019). In China, such removal is carried out manually to maintain visually tidy green spaces, a practice that increases labor costs while reducing biodiversity and ecosystem functioning (Zhao et al. 2025). As urbanization accelerates, human–nature interactions increasingly occur within cities, making urban areas critical for future biodiversity conservation (Crane and Kinzig 2005; Spotswood et al. 2021). In this context, incorporating spontaneous herbaceous vegetation into urban green spaces offers a cost‐effective way to enhance biodiversity and climate resilience (Chen et al. 2021; Li, Zhang, et al. 2023).

Accordingly, approaches such as urban rewilding and nature‐based solutions emphasize vegetation complexity and heterogeneity to support biodiversity in cities (Chen and Wang 2025). Empirical evidence shows that areas containing spontaneous herbaceous vegetation support higher plant and invertebrate diversity than intensively managed lawns (Robinson and Lundholm 2012), while more diverse plant assemblages can suppress invasive species and enhance ecosystem functioning (Prach and Walker 2011). At the microbial scale, heterogeneous herbaceous communities can foster more complex microbiomes, with potential benefits for human health (Mills et al. 2019). From a social perspective, urban residents increasingly accept—and often prefer—landscapes that integrate spontaneous and cultivated vegetation (Muratet et al. 2015).

Urban green spaces are typically dominated by a limited number of cultivated vegetation types, such as turfgrass lawns and ornamental groundcovers, which differ markedly in vegetation structure, resource availability, and management intensity. These differences represent distinct planting designs that shape microenvironmental conditions and influence the establishment, composition, and persistence of spontaneous plant communities. Accordingly, approaches such as urban rewilding and nature‐based solutions emphasize vegetation complexity and heterogeneity to support biodiversity in cities (Chen and Wang 2025). Empirical evidence shows that areas containing spontaneous herbaceous vegetation support higher plant and invertebrate diversity than intensively managed lawns (Robinson and Lundholm 2012), while more diverse plant assemblages can suppress invasive species and enhance ecosystem functioning (Prach and Walker 2011). At the microbial scale, heterogeneous herbaceous communities can foster more complex microbiomes, with potential benefits for human health (Mills et al. 2019). From a social perspective, urban residents increasingly accept—and often prefer—landscapes that integrate spontaneous and cultivated vegetation (Muratet et al. 2015).

Residential green spaces constitute the most widespread urban landscape patches and are the most accessible green spaces for daily use (Yang et al. 2019). They provide recreational and social benefits while serving as important habitats for spontaneous herbaceous plant communities (Southon et al. 2017). The diversity of spontaneous herbaceous plant communities enhances visual quality and helps alleviate psychological stress and anxiety among residents (De la Barrera et al. 2016; Correa et al. 2021). However, management practices and planting preferences can lead to contrasting ecological outcomes. Studies of residential lawns across the United States show that simplified landscaping preferences and intensive management practices consistently reduce plant diversity, thereby accelerating biotic homogenization and eroding ecological functions (Blanchette et al. 2021; Wheeler et al. 2017; Russo et al. 2024). In contrast, several European countries have incorporated native spontaneous herbs into residential green spaces, enhancing biodiversity and improving microclimate regulation (Jamei et al. 2019; Langemeyer et al. 2021; Segar et al. 2022). These differences reflect contrasting governance systems. In many Western countries, urban green spaces are often managed at the household level, whereas in China they are governed by centralized property management systems (Yan et al. 2019), which limits the direct transferability of international experience.

Early studies of spontaneous herbaceous plants focused primarily on species richness and diversity patterns in specific urban habitats, such as vacant lots, sidewalks, and green roofs (Schwoertzig et al. 2016; Blouin et al. 2019). More recent research has shifted toward identifying the drivers of species composition and diversity across spatial scales (Qian et al. 2020; Hu et al. 2022). However, the mechanisms shaping spontaneous herbaceous plant communities at fine spatial scales remain poorly understood, particularly the role of cultivated vegetation introduced through landscape design (Chen et al. 2025). Moreover, while diversity patterns are increasingly documented, their implications for community stability remain debated, especially in relation to how different components of diversity contribute to the regulation of community dynamics.

This study investigates how cultivated vegetation influences the composition, diversity, and stability of spontaneous herbaceous plant communities in residential green spaces. Specifically, we address three questions: (1) how do different types of cultivated vegetation affect the composition and diversity of spontaneous herbaceous species? (2) does seasonal variation in species richness and diversity differ among vegetation types? and (3) how does cultivated vegetation influence the dissimilarity and stability of spontaneous plant communities?

Based on differences in vegetation structure and management intensity, we expect that more homogeneous and intensively managed vegetation types will impose stronger environmental filtering, reducing overall diversity and disproportionately constraining dominant species. In contrast, more heterogeneous planting configurations may provide a wider range of microsites, facilitating the persistence of occasional species and enhancing community stability. To test these expectations, we conducted field surveys across 21 residential green spaces in a medium‐sized city in China, quantifying the structure and diversity of spontaneous herbaceous plant communities under different cultivated vegetation types.

## 2 Material and Methods

### 2.1 Study Area

**FIGURE 1:**
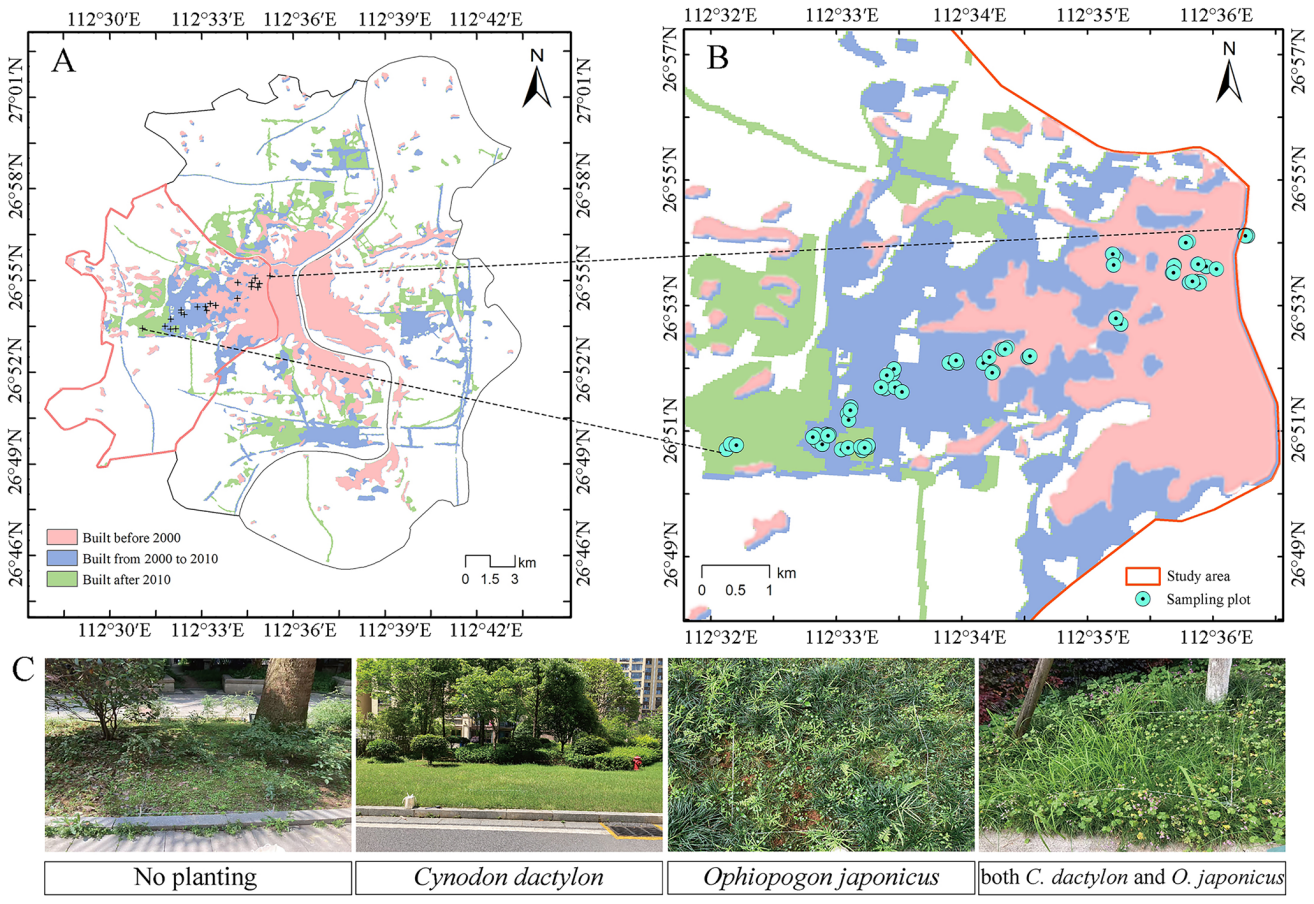
Study area and sampling plots. The map data was obtained from the National Platform for common GeoSpatial Information Services (https://www.tianditu.gov.cn/).

This study was conducted in Zhengxiang District, the urban core of Hengyang City, South China (112°28′27′′–112°36′00′′E, 26°48′27′′–26°57′39′′N; Figure 1A). The area features flat terrain traversed by the Xiangjiang River and a subtropical monsoon climate with distinct seasons, with a mean annual temperature of approximately 19.4°C. By the end of 2024, the built‐up area of Hengyang's central urban zone had reached 144 km^2^, supporting a permanent population of approximately 1.36 million.

As the political, economic, and cultural center of Hengyang, Zhengxiang District has undergone rapid urbanization in recent decades, resulting in substantial loss of native vegetation. Residential developments spanning different historical periods reflect common urbanization patterns observed in medium‐sized cities and small towns throughout China. Together, these characteristics make Zhengxiang District a suitable case study for examining the ecological processes shaping the composition and distribution of spontaneous herbaceous plant communities in residential green spaces.

### 2.2 Sampling Design

Twenty‐one residential green spaces in Zhengxiang District, established during three phases of urban development (before 2000, 2000–2010, and after 2010), were selected (Figure 1B). Within each site, surveys were conducted across a range of microhabitat types, including lawns, crevices, flower beds, tree pits, understory areas, shrub edges, and grass‐brick plantings. The number of plots per site was determined using a species accumulation approach (species–sample number curve; Zhao et al. 2023). Sampling was considered sufficient when the number of newly recorded species in an additional plot was ≤ 10% of that observed in the preceding plot. Based on this criterion, 3–8 plots (1 m × 1 m quadrat) were established per site. The variation in plot number reflects differences in site size and habitat heterogeneity, with more heterogeneous sites requiring a greater number of plots to capture species diversity.

Field surveys were conducted in both spring and autumn, with two sampling rounds in each season. In total, 285 sampling points were surveyed (148 in spring and 137 in autumn), and plot locations were kept consistent across sampling periods to allow direct seasonal comparisons. Following field surveys, plots were classified into four vegetation types based on the presence and combination of two dominant cultivated species, *Cynodon dactylon* and *Ophiopogon japonicus* : P1 (no cultivated species), P2 ( *C. dactylon* only), P3 ( *O. japonicus* only), and P4 (both species present) (Figure 1C). This classification was based on observed vegetation composition rather than experimental manipulation.

### 2.3 Data Collection

Field surveys were conducted in April–May and September–October. Within each plot, herbaceous plant data (including both cultivated and spontaneous species) were recorded, including species identity, abundance, and percentage cover. Taxonomic information, including family, genus, life form, and species source, was verified using the Flora of Hunan (Liu 2000) and the Plant Science Data Center (https://www.iplant.cn/). Abundance was defined as the number of individuals of each species recorded within each quadrat. Frequency was defined as the number of quadrats in which a given species occurred within each site, and relative frequency was calculated as the proportion of quadrats occupied by each species within the total number of quadrats surveyed. Percentage cover was visually estimated as the proportion of ground surface occupied by each species within a plot, following standard ecological survey protocols (Kent 2011). To improve consistency, cover estimates were made by the same trained observers across all surveys. Based on these data, spontaneous herbaceous species were identified and distinguished from cultivated species within each plot. A complete list of recorded spontaneous herbaceous species is provided in Table S1.

### 2.4 Statistical Analyses

Statistical analyses were conducted to quantify the composition, diversity, and stability of spontaneous herbaceous communities across vegetation types, with plots lacking cultivated vegetation (P1) treated as controls. Species were classified into three groups (dominant, common, and occasional) based on importance values (IV), calculated as the average of relative abundance, relative frequency, and relative cover. Species with IV > 10%, 5%–10%, and < 5% were classified as dominant, common, and occasional species, respectively. K‐means clustering was applied to support and validate this classification.

Species composition was compared using a G‐test (goodness‐of‐fit test) with equal occurrence as the null hypothesis. Species richness and diversity were assessed using the Margalef index and the Shannon–Wiener index. Community compositional dissimilarity among plots was evaluated using the Bray–Curtis index. Community stability was quantified using the Average Variance Degree (AVD) index, which reflects the temporal variability of species' relative abundances within the community. Lower AVD values indicate lower variability and thus higher stability (Xun et al. 2021; Xu et al. 2022). This metric was selected because it captures fluctuations in overall community structure rather than focusing on individual species persistence, making it suitable for evaluating stability at the community‐level.

Seasonal differences between spring and autumn were assessed using generalized linear models (GLMs) with normal distribution and an identity link function (the dependent variable is not transformed). Differences among vegetation types were tested using one‐way analysis of variance (ANOVA) followed by Duncan's multiple range test in IBM SPSS Statistics 25.0. Pearson correlation analysis was conducted in R (version 4.3.2) to examine relationships among diversity, dissimilarity, and stability indices. Data preprocessing was performed in Microsoft Excel 2010, and figures were generated using Origin 2022.

## 3 Results

### 3.1 Species Composition of Spontaneous Herbaceous Communities

Across all sampling plots, 145 spontaneous herbaceous species were recorded, belonging to 121 genera and 53 families (Table S1). Asteraceae (20 species) and Poaceae (11 species) were the most species‐rich families. In terms of life form, 73 (50.34%) were annuals, 67 (46.20%) perennials, and 5 (3.45%) biennials. In terms of source, 99 species (68.28%) were native, whereas 46 (31.72%) were non‐native. Seasonally, 113 species were recorded in spring and 98 species in autumn, with no significant difference in species richness between seasons (G = 0.93, *p* = 0.34). Across vegetation types, species richness did not differ significantly between plots planted with *Cynodon dactylon* (P2: 99 species) and control plots without cultivation (P1: 116 species) (G = 1.19, *p* = 0.28). In contrast, plots planted with *Ophiopogon japonicus* (P3: 44 species) and mixed vegetation (P4: 26 species) supported significantly fewer species than the control (P1) (G_1_ = 32.63, *p*_1_ < 0.001; G_2_ = 60.17, *p*_2_ < 0.001).

Nine dominant species were identified, all occurring consistently across plots, including four native perennials and five annuals (Table 1). Common species (17) were mainly native and widely distributed. In contrast, occasional species (119) comprised the majority of taxa and showed patchy distributions, with many restricted to a single season (Table S1). These species were primarily concentrated in P1 and P2, with a few recorded numbers. Overall, dominant and common species showed broad spatial persistence, whereas occasional species were more spatially and temporally restricted.

**TABLE 1:** List of dominant spontaneous herbaceous species recorded in the study, including taxonomic information, life form, source, growing season, and vegetation type, and importance values.

Species	Family	Life form	Source	Season	Vegetation type^1^	Importance value
*Acalypha australis*	Euphorbiaceae	Annual	Native	Spring & Autumn	P1/P2/P3/P4	12.65%
Clinopodium chinense	Lamiaceae	Perennial	Native	Spring & Autumn	P1/P2/P3/P4	26.75%
*Hydrocotyle sibthorpioides*	Araliaceae	Perennial	Native	Spring & Autumn	P1/P2/P3/P4	11.52%
*Youngia japonica*	Asteraceae	Annual	Native	Spring & Autumn	P1/P2/P3/P4	17.06%
*Oxalis corniculata*	Oxalidaceae	Perennial	Native	Spring & Autumn	P1/P2/P3/P4	25.97%
*Phyllanthus urinaria*	Phyllanthaceae	Annual	Native	Spring & Autumn	P1/P2/P3/P4	20.19%
*Poa annua*	Poaceae	Annual	Native	Spring & Autumn	P1/P2/P3/P4	13.59%
*Stellaria media*	Caryophyllaceae	Annual	Native	Spring & Autumn	P1/P2/P3/P4	11.84%
*Viola philippica*	Violaceae	Perennial	Native	Spring & Autumn	P1/P2/P3/P4	13.03%

*Note:* Vegetation types^1^: P1, no cultivated vegetation; P2, *Cynodon dactylon* ; P3, *Ophiopogon japonicus* ; P4, mixed vegetation.

### 3.2 Effects of Cultivated Vegetation on Species Diversity

**FIGURE 2:**
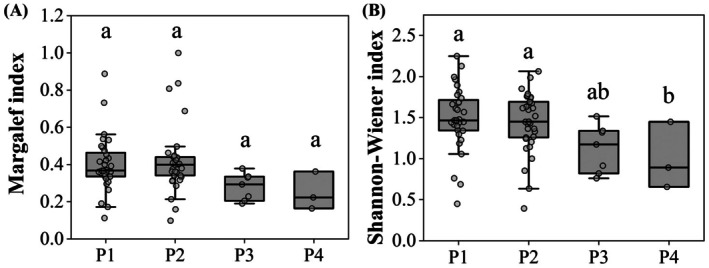
Species richness (A) and Shannon–Wiener diversity (B) across four vegetation types. Different lowercase letters indicate significant differences. Vegetation types: P1, no cultivated vegetation; P2, *Cynodon dactylon* ; P3, *Ophiopogon japonicus* ; P4, mixed vegetation.

Species richness did not differ significantly among vegetation types (*F* = 2.350, *p* = 0.080). In contrast, the Shannon–Wiener diversity index varied significantly across vegetation types (*F* = 3.218, *p* = 0.028; Figure 2). Higher diversity values were observed in plots without cultivated vegetation (P1) and those dominated by *C. dactylon* (P2), whereas plots dominated by *O. japonicus* (P3) and mixed vegetation (P4) exhibited comparatively lower diversity. Although species richness did not differ significantly, it followed a similar pattern, with higher values in P1 and P2 and lower values in P3 and P4.

### 3.3 Seasonal Variation in Spontaneous Herbaceous Plant Diversity

**FIGURE 3:**
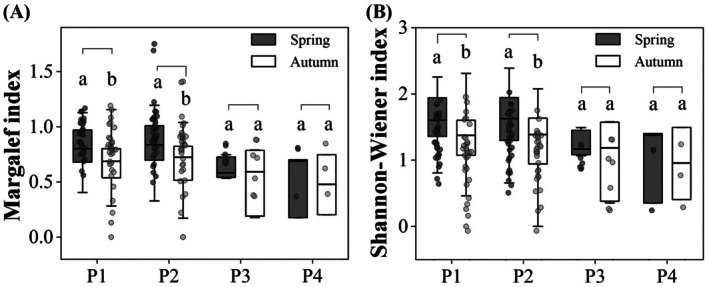
Seasonal variation (spring vs. autumn) in species richness (A) and Shannon–Wiener diversity (B) across four vegetation types. Different lowercase letters indicate significant differences. Vegetation types: P1, no cultivated vegetation; P2, *Cynodon dactylon* ; P3, *Ophiopogon japonicus* ; P4, mixed vegetation.

Seasonal patterns of species diversity varied among vegetation types (Figure 3). In plots without cultivated vegetation (P1) and those dominated by *C. dactylon* (P2), both the Margalef richness index and the Shannon–Wiener diversity index were significantly higher in spring than in autumn (P1: Margalef χ^2^ = 6.242, *p* = 0.012; Shannon–Wiener χ^2^ = 6.862, *p* = 0.009; P2: Margalef χ^2^ = 7.501, *p* = 0.006; Shannon–Wiener χ^2^ = 7.562, *p* = 0.006). In contrast, no significant seasonal differences were detected in plots dominated by *O. japonicus* (P3) and mixed vegetation (P4) (P3: Margalef χ^2^ = 3.333, *p* = 0.068; Shannon–Wiener χ^2^ = 3.119, *p* = 0.077; P4: Margalef χ^2^ = 1.399, *p* = 0.237; Shannon–Wiener χ^2^ = 1.113, *p* = 0.291). Although species richness and Shannon–Wiener diversity did not differ significantly among vegetation types either in spring (Margalef *F* = 0.500; *p* = 0.683; Shannon–Wiener *F* = 0.785; *p* = 0.503) or autumn (Margalef *F* = 1.424; *p* = 0.482; Shannon–Wiener *F* = 1.109; *p* = 0.268), they followed a similar pattern, with higher values in P1 and P2 than in P3 and P4.

### 3.4 Community Dissimilarity and Stability Across Vegetation Types

**FIGURE 4:**
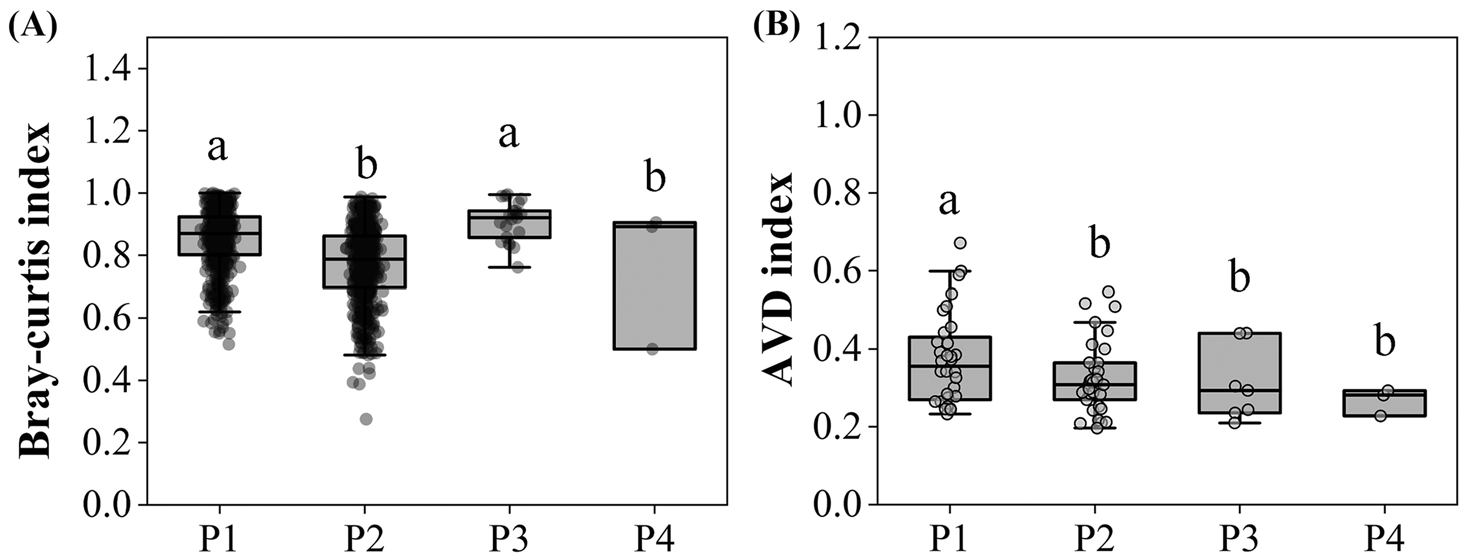
Community dissimilarity (A) and community stability (B) across four vegetation types. Different lowercase letters indicate significant differences. Vegetation types: P1, no cultivated vegetation; P2, *Cynodon dactylon* ; P3, *Ophiopogon japonicus* ; P4, mixed vegetation.

**FIGURE 5:**
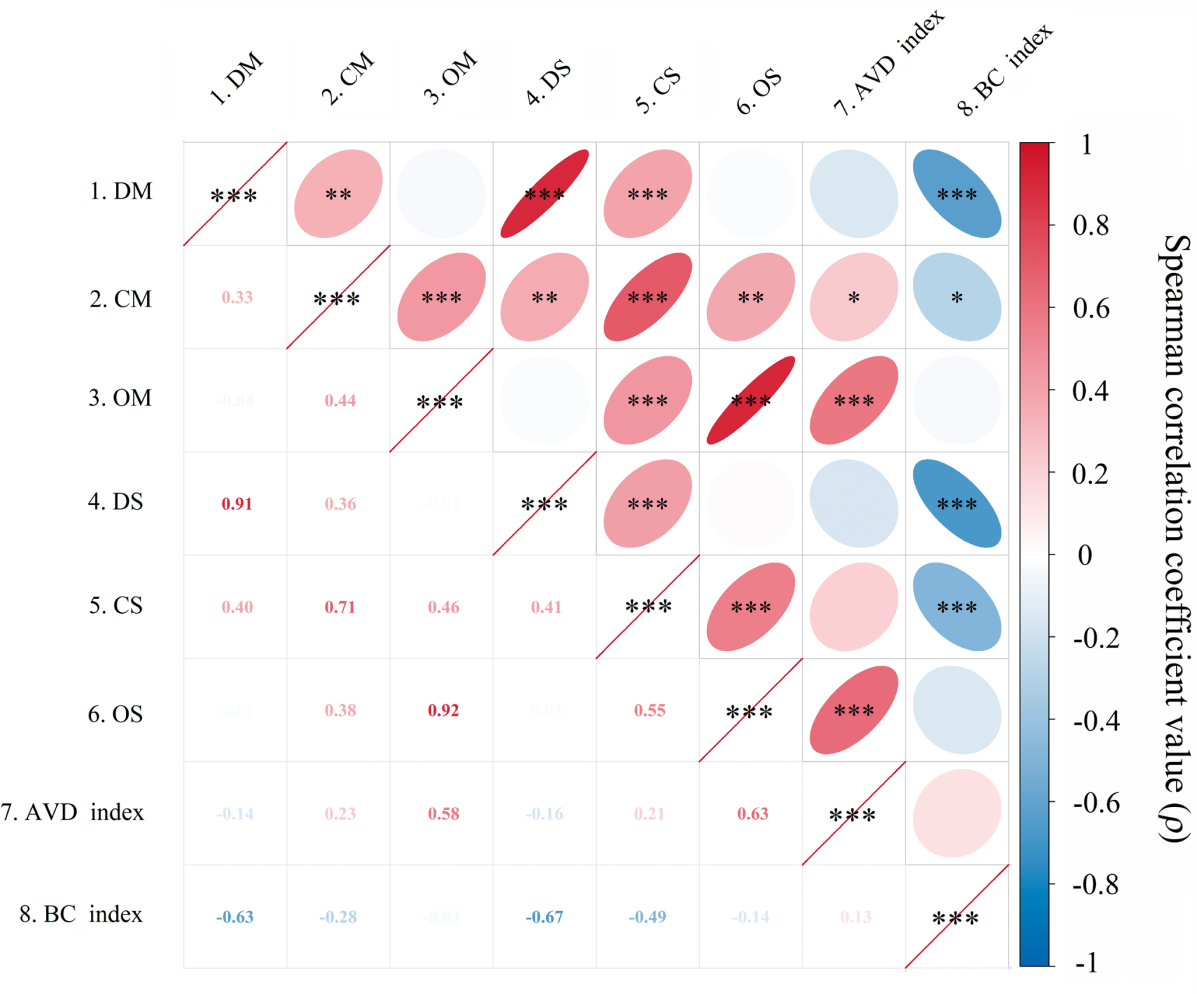
1–3. DM, CM, and OM denote the mean abundance of dominant, common, occasional species. 4–6. DS, CS, and OS denote species richness of dominant, common, and occasional species, respectively. 7. Average variation degree index. 8. Bray‐Curtis index. *p < 0.05, **p < 0.01, ***p < 0.001.

Community dissimilarity and stability varied significantly across vegetation types (dissimilarity: *F* = 9.561, *p* < 0.001; stability: *F* = 3.139, *p* = 0.031; Figure 4). The Bray–curtis index was lower in P2 than P1 (χ^2^ = 115.607, *p* < 0.001), while no significant difference between plots dominated by P3 and P1 (χ^2^ = 5.279, *p* = 0.022), indicating Plots dominated by *C. dactylon* (P2) has lower community dissimilarity than plots dominated by *O. japonicus* (P3). All of the AVD index in P2, P3 and P4 lower than control (P1), indicating cultivated vegetation adverse to community stability (all *p* < 0.05). Correlation analysis further revealed that community dissimilarity was significantly negatively associated with the richness and diversity of dominant and common species (all *p* < 0.05; Figure 5). In contrast, community stability was significantly positively associated with the richness and diversity of occasional species (both *p* < 0.001; Figure 5).

## 4 Discussion

### 4.1 Urban Residential Green Spaces as Biodiversity Reservoirs Under Anthropogenic Filtering

Urban residential green spaces can support high levels of spontaneous herbaceous plant diversity and distinct species composition despite intensive human management. In this study, a total of 145 spontaneous herbaceous species were recorded, of which native species accounted for 68.28%. The species richness of spontaneous herbaceous plants in Hengyang exceeded the national median reported across 59 Chinese cities (Hu et al. 2022), indicating that residential green spaces can function as important reservoirs of urban biodiversity. The relatively high proportion of native species suggests that residential green spaces retain elements of local flora and contribute to the conservation of regional biodiversity. These findings highlight the ecological value of spontaneous plant communities, which have increasingly been recognized as important components of urban ecosystems that support biodiversity and human well‐being (Robinson and Lundholm 2012; Nizamani et al. 2023). Previous studies have primarily emphasized large urban green spaces, such as parks and forests, as key components of urban species pools (Exantus et al. 2021; Toledo‐Garibaldi et al. 2023), while the contribution of spontaneous vegetation in residential areas has received comparatively less attention. Our results suggest that residential green spaces, although smaller and more intensively managed, can make a substantial contribution to urban biodiversity.

The observed taxonomic composition, dominated by Asteraceae and Poaceae and characterized by a high proportion of annual species, is consistent with patterns reported in other urban ecosystems, where disturbance‐tolerant and fast‐colonizing species prevail (Cervelli et al. 2013; Qiu et al. 2021; Li, Zhang, et al. 2023). This pattern reflects the combined influence of anthropogenic disturbance and environmental filtering in shaping spontaneous plant communities at fine spatial scales.

### 4.2 Effects of Cultivated Vegetation on Spontaneous Herbaceous Species Diversity

Comparative analyses showed that plots containing cultivated vegetation (P2–P4) generally supported lower diversity of spontaneous herbaceous plants than plots without cultivated species (P1), indicating an overall suppressive effect of cultivated vegetation. This pattern is consistent with previous studies showing that cultivated vegetation and associated management practices can modify resource availability and competitive environments, thereby shaping spontaneous plant assemblages (Omar et al. 2018; Gao et al. 2021; Chen et al. 2025). However, the magnitude of this effect varied among vegetation types, highlighting the importance of vegetation structure and species‐specific characteristics. In plots dominated by *Cynodon dactylon* (P2), the diversity of dominant and common spontaneous species remained comparable to that in control plots, while occasional species showed only a slight reduction. This suggests that *C. dactylon* lawns impose relatively moderate constraints on spontaneous plant establishment. A possible explanation is that *C. dactylon* forms a relatively low and less continuously closed canopy structure, which allows partial light penetration and maintains a greater number of microsites for germination. Such structural openness may reduce the intensity of aboveground competition and facilitate coexistence with spontaneous species under moderate management conditions (Deparis et al. 2023). In contrast, plots dominated by *Ophiopogon japonicus* (P3) exhibited consistently lower diversity across all spontaneous species groups. This pattern is likely associated with its dense and compact growth form, which reduces light availability at the soil surface and limits available microsites for establishment. In addition, its dense rhizome system may enhance belowground competition by increasing resource uptake efficiency, thereby reducing water and nutrient availability for co‐occurring species (Casper and Jackson 1997; Cahill Jr and McNickle 2011; Heuermann et al. 2019). Moreover, ornamental groundcover systems are typically subject to relatively intensive maintenance, which can further reduce environmental heterogeneity and limit spontaneous species recruitment (Walsh and Allan 2023). Together, these factors suggest that the lower diversity observed in *O. japonicus* plots is driven by the combined effects of vegetation structure and management intensity, rather than a single dominant mechanism. Overall, these results indicate that cultivated vegetation does not uniformly suppress spontaneous herbaceous plants; instead, its effects depend on species traits, vegetation structure, and associated management practices.

### 4.3 Diversity Structure and Community Stability of Spontaneous Plant Communities

Our results show that cultivated vegetation influences community stability primarily through changes in species frequency structure rather than overall species richness. In particular, overall species diversity was negatively associated with community stability, indicating that higher diversity does not necessarily enhance stability in these managed systems and may instead be linked to greater variability in community‐level abundance patterns (McKinney 2006; Groffman et al. 2014). In contrast, the diversity of occasional species was positively associated with community stability. This result highlights the importance of species frequency structure in shaping stability patterns and is consistent with previous studies showing that urban spontaneous vegetation is typically characterized by a few widespread species and many low‐frequency species with restricted distributions (Cervelli et al. 2013; Qian et al. 2020). One possible explanation is that occasional species, although individually rare, may exhibit asynchronous occurrence patterns across plots, thereby reducing variability in aggregate community properties. Similar dynamics have been reported in fragmented urban habitats, where species persistence reflects a balance between local extinction and recolonization processes (Piano et al. 2020). However, this interpretation remains correlative and requires further experimental validation. Overall, these findings suggest that community stability in spontaneous herbaceous systems is more closely associated with species frequency structure than with total species richness, emphasizing the importance of incorporating frequency‐based metrics into analyses of urban plant communities.

### 4.4 Implications for Spontaneous Plant Conservation and Landscape Design in Residential Areas

Our results suggest that cultivated vegetation influences spontaneous herbaceous communities primarily through changes in species frequency structure and habitat conditions, with important implications for the design and management of residential green spaces. Rather than replacing cultivated vegetation, a more effective strategy is to adjust planting configuration and management intensity to facilitate coexistence between spontaneous and cultivated species. Reducing planting density and increasing structural heterogeneity can create a wider range of microsites, thereby promoting the establishment and persistence of spontaneous species (Thompson and McCarthy 2008). Such approaches align with growing recognition that spontaneous herbaceous species can contribute to novel urban biodiversity assemblages and ecosystem functioning in low‐maintenance systems (Del Tredici 2010; Li, Zhang, et al. 2023; Yang et al. 2023). Vegetation identity also plays a key role. Our results indicate that *C. dactylon* shows relatively high compatibility with spontaneous species, particularly by maintaining a more diverse assemblage of occasional species. This suggests that selecting cultivated species with less competitive growth forms or lower canopy closure may enhance spontaneous species recruitment (Stewart et al. 2009; Ignatieva et al. 2015). Management practices further influence spontaneous species dynamics. Intensive maintenance associated with ornamental groundcovers tends to increase vegetation uniformity and reduce establishment opportunities, whereas reduced mowing or selective thinning may alleviate competition and enhance habitat heterogeneity (Yang et al. 2022). Mixed‐species plantings introduce additional complexity, but their effects are context‐dependent. Some species combinations may intensify competition and reduce opportunities for low‐frequency species, whereas mixtures with complementary traits may better support biodiversity and functional stability (Shi et al. 2020). Overall, these findings highlight the importance of integrating vegetation composition, structural characteristics, and management regimes in residential green space design. Managing for heterogeneity, rather than uniformity, is key to supporting coexistence and enhancing the ecological value of urban landscapes.

## 5 Conclusions

Cultivated vegetation is a key driver shaping the composition, diversity, and stability of spontaneous herbaceous communities in residential green spaces. Its effects are non‐uniform and vary across vegetation types and species groups defined by importance values. Across vegetation types, cultivated planting generally reduced the diversity of spontaneous herbaceous species, with particularly strong effects on occasional species, leading to shifts in species frequency structure. In contrast, dominant species showed relatively consistent occurrence, indicating greater tolerance to environmental filtering associated with cultivated vegetation. Seasonal variation in diversity further depended on vegetation type, with significant spring‐autumn differences observed only in less intensively managed plots, suggesting that vegetation structure and management can mediate temporal dynamics of spontaneous communities. Community dissimilarity and stability were both closely associated with species frequency structure rather than overall species richness. Dissimilarity decreased with increasing diversity of dominant and common species, whereas community stability was significantly related to the diversity of occasional species, indicating that different components of species frequency contribute differently to community patterns. Among the vegetation types examined, *Cynodon dactylon* showed relatively high compatibility with spontaneous species, whereas *Ophiopogon japonicus* and mixed planting imposed stronger constraints on diversity. Overall, these findings highlight the importance of vegetation composition, structural characteristics, and management regimes in shaping spontaneous plant communities. Managing for moderate heterogeneity, rather than uniformity, may help promote coexistence and enhance the ecological value of urban residential green spaces.

## Author Contributions

**Jia Peng:** conceptualization (equal), data curation (equal), formal analysis (equal), investigation (equal), methodology (equal), software (equal), writing – original draft (equal). **Jing Liu:** conceptualization (equal), data curation (equal), formal analysis (equal), investigation (equal), methodology (equal), validation (equal), writing – original draft (equal). **Zhihuan Huang:** conceptualization (equal), funding acquisition (lead), methodology (equal), project administration (lead), supervision (lead), writing – original draft (equal), writing – review and editing (lead).

## Funding

This research was funded by National Natural Science Foundation of China (32471596, 31700196); Natural Science Foundation of Hunan Province (2020JJ5477).

## Conflicts of Interest

The authors declare no conflicts of interest.

## Supporting information


**Table S1:** List of spontaneous herbaceous species recorded in the study, including taxonomic information, life form, source, growing season, and vegetation type.

## Data Availability

The datasets generated and/or analyzed during the current study are available at https://doi.org/10.6084/m9.figshare.30252214.
