# Supplementary material for: Cultivated Vegetation Shapes Diversity and Stability of Spontaneous Herbaceous Communities in Residential Green Spaces
Source: Ecol Evol. 2026 May 30;16(6):e73761. doi: 10.1002/ece3.73761 (PMC13239907; doi:10.1002/ece3.73761)
Supplement: Supplementary file 1 — Table S1: List of spontaneous herbaceous species recorded in the study, including taxonomic information, life form, source, growing season, and vegetation type. [file ECE3-16-e73761-s001.docx]

**Table S1.** List of spontaneous herbaceous species recorded in the study, including taxonomic information, life form, source, growing season, and vegetation type.

| **Species** | **Family** | **Life form** | **Source** | **Season** | **Vegetation type^1^** |
| --- | --- | --- | --- | --- | --- |
| *Justicia procumbens* | Acanthaceae | Annual | Native | Autumn | P2 |
| *Acorus calamus* | Acoraceae | Perennial | Native | Autumn | P1/P2 |
| *Achyranthes aspera* | Amaranthaceae | Perennial | Native | Spring & Autumn | P1/P2 |
| *Alternanthera philoxeroides* | Amaranthaceae | Perennial | Alien | Spring & Autumn | P1/P2/P3 |
| *Chenopodium album* | Amaranthaceae | Annual | Native | Autumn | P1 |
| *Allium giganteum* | Amaryllidaceae | Perennial | Alien | Autumn | P1 |
| *Zephyranthes carinata* | Amaryllidaceae | Perennial | Alien | Spring & Autumn | P1/P4 |
| *Centella asiatica* | Apiaceae | Perennial | Native | Spring & Autumn | P1/P2 |
| *Daucus carota* | Apiaceae | Biennial | Alien | Spring & Autumn | P1/P2 |
| *Torilis scabra* | Apiaceae | Annual | Native | Spring | P1/P2/P3 |
| *Aglaonema modestum* | Araceae | Perennial | Alien | Autumn | P1 |
| *Alocasia odora* | Araceae | Perennial | Native | Spring & Autumn | P1 |
| *Pinellia cordata* | Araceae | Perennial | Native | Spring | P1 |
| *Pinellia ternata* | Araceae | Perennial | Native | Spring | P2 |
| *Typhonium blumei* | Araceae | Perennial | Native | Spring & Autumn | P1 |
| *Hydrocotyle sibthorpioides* | Araliaceae | Perennial | Native | Spring & Autumn | P1/P2/P3/P4 |
| *Hydrocotyle verticillata* | Araliaceae | Perennial | Alien | Spring & Autumn | P1 |
| *Agave sisalana* | Asparagaceae | Perennial | Alien | Autumn | P1 |
| *Asparagus cochinchinensis* | Asparagaceae | Perennial | Native | Spring | P2 |
| *Chlorophytum comosum* | Asparagaceae | Perennial | Alien | Spring | P1 |
| *Ophiopogon japonicus* | Asparagaceae | Perennial | Native | Spring & Autumn | P1/P2 |
| *Artemisia annua* | Asteraceae | Annual | Native | Spring | P2 |
| *Artemisia argyi* | Asteraceae | Perennial | Native | Autumn | P1 |
| *Bidens pilosa* | Asteraceae | Annual | Alien | Spring | P3 |
| *Callistephus chinensis* | Asteraceae | Annual | Alien | Spring | P1 |
| *Centipeda minima* | Asteraceae | Annual | Native | Autumn | P2 |
| *Cirsium japonicum* | Asteraceae | Perennial | Native | Spring & Autumn | P1 |
| *Eclipta prostrata* | Asteraceae | Annual | Native | Autumn | P2 |
| *Erigeron annuus* | Asteraceae | Annual | Alien | Spring & Autumn | P1/P2/P3/P4 |
| *Erigeron bonariensis* | Asteraceae | Annual | Alien | Autumn | P3 |
| *Erigeron canadensis* | Asteraceae | Annual | Alien | Spring & Autumn | P1/P2/P3/P4 |
| *Erigeron philadelphicus* | Asteraceae | Annual | Alien | Spring | P2 |
| *Erigeron sumatrensis* | Asteraceae | Annual | Alien | Spring & Autumn | P1/P2/P3 |
| *Ixeris polycephala* | Asteraceae | Annual | Native | Spring | P2 |
| *Lactuca serriola* | Asteraceae | Annual | Alien | Spring & Autumn | P1/P2/P4 |
| *Lapsanastrum apogonoides* | Asteraceae | Annual | Native | Spring | P1/P2 |
| *Pseudognaphalium affine* | Asteraceae | Annual | Native | Spring & Autumn | P1/P2 |
| *Sonchus oleraceus* | Asteraceae | Annual | Native | Spring & Autumn | P1/P2 |
| *Symphyotrichum subulatum* | Asteraceae | Annual | Alien | Spring & Autumn | P1/P2/P3/P4 |
| *Taraxacum mongolicum* | Asteraceae | Perennial | Native | Spring | P1/P2 |
| *Youngia japonica* | Asteraceae | Annual | Native | Spring & Autumn | P1/P2/P3/P4 |
| *Reboulia hemisphaerica* | Aytoniaceae | Perennial | Native | Autumn | P1 |
| *Bothriospermum zeylanicum* | Boraginaceae | Annual | Native | Spring | P2 |
| *Trigonotis peduncularis* | Boraginaceae | Annual | Native | Spring & Autumn | P1/P2 |
| *Arabidopsis thaliana* | Brassicaceae | Annual | Native | Autumn | P1 |
| *Capsella bursa-pastoris* | Brassicaceae | Annual | Native | Spring | P1/P2 |
| *Cardamine occulta* | Brassicaceae | Annual | Native | Spring & Autumn | P1/P2/P3 |
| *Lepidium didymum* | Brassicaceae | Annual | Alien | Spring | P1/P2/P3 |
| *Nasturtium officinale* | Brassicaceae | Perennial | Alien | Autumn | P1 |
| *Orychophragmus violaceus* | Brassicaceae | Annual | Native | Spring | P1/P2 |
| *Rorippa indica* | Brassicaceae | Annual | Native | Spring & Autumn | P1/P3 |
| *Campanula medium* | Campanulaceae | Biennial | Alien | Autumn | P2 |
| *Triodanis perfoliata* | Campanulaceae | Annual | Alien | Spring | P2 |
| *Wahlenbergia marginata* | Campanulaceae | Perennial | Native | Autumn | P2 |
| *Cerastium glomeratum* | Caryophyllaceae | Annual | Alien | Spring & Autumn | P1/P2/P3/P4 |
| *Drymaria cordata* | Caryophyllaceae | Annual | Native | Spring | P1/P2 |
| *Sagina japonica* | Caryophyllaceae | Annual | Native | Spring | P1/P2/P4 |
| *Stellaria media* | Caryophyllaceae | Annual | Native | Spring & Autumn | P1/P2/P3/P4 |
| *Commelina communis* | Commelinaceae | Annual | Native | Spring | P1/P2 |
| *Calystegia hederacea* | Convolvulaceae | Annual | Native | Autumn | P2 |
| *Phedimus aizoon* | Crassulaceae | Perennial | Alien | Spring & Autumn | P1 |
| *Sedum bulbiferum* | Crassulaceae | Perennial | Native | Spring & Autumn | P1/P2 |
| *Sedum emarginatum* | Crassulaceae | Perennial | Native | Spring | P1 |
| *Sedum lineare* | Crassulaceae | Perennial | Native | Spring & Autumn | P1/P2 |
| *Melothria pendula* | Cucurbitaceae | Annual | Alien | Autumn | P3 |
| *Fimbristylis littoralis* | Cyperaceae | Annual | Native | Autumn | P2 |
| *Kyllinga brevifolia* | Cyperaceae | Perennial | Native | Autumn | P2/P3 |
| *Acystopteris japonica* | Cystopteridaceae | Perennial | Native | Spring | P1 |
| *Acalypha australis* | Euphorbiaceae | Annual | Native | Spring & Autumn | P1/P2/P3/P4 |
| *Euphorbia maculata* | Euphorbiaceae | Annual | Alien | Autumn | P1/P2 |
| *Euphorbia prostrata* | Euphorbiaceae | Annual | Alien | Autumn | P1 |
| *Astragalus sinicus* | Fabaceae | Biennial | Native | Spring & Autumn | P1 |
| *Kummerowia striata* | Fabaceae | Annual | Native | Spring & Autumn | P2 |
| *Senna obtusifolia* | Fabaceae | Annual | Alien | Spring & Autumn | P1/P2 |
| *Trifolium repens* | Fabaceae | Perennial | Alien | Spring & Autumn | P1/P2/P3/P4 |
| *Geranium carolinianum* | Geraniaceae | Annual | Alien | Spring & Autumn | P1/P2 |
| *Hypericum japonicum* | Hypericaceae | Annual | Native | Spring & Autumn | P1/P2 |
| *Clinopodium chinense* | Lamiaceae | Perennial | Native | Spring & Autumn | P1/P2/P3/P4 |
| *Mentha arvensis* | Lamiaceae | Perennial | Native | Spring & Autumn | P1/P2 |
| *Perilla frutescens* | Lamiaceae | Annual | Native | Spring & Autumn | P1 |
| *Pogostemon cablin* | Lamiaceae | Perennial | Alien | Autumn | P1 |
| *Linum usitatissimum* | Linaceae | Annual | Alien | Spring | P1/P2/P3 |
| *Lygodium japonicum* | Lygodiaceae | Perennial | Native | Spring & Autumn | P1/P2/P3 |
| *Torenia concolor* | Linderniaceae | Perennial | Native | Autumn | P1/P2 |
| *Alcea rosea* | Malvaceae | Biennial | Native | Spring | P1 |
| *Mazus pumilus* | Mazaceae | Annual | Native | Spring & Autumn | P1/P2/P4 |
| *Trigastrotheca stricta* | Molluginaceae | Annual | Native | Autumn | P1 |
| *Fatoua villosa* | Moraceae | Annual | Native | Spring & Autumn | P1/P2/P3 |
| *Mirabilis jalapa* | Nyctaginaceae | Perennial | Alien | Spring & Autumn | P1 |
| *Epilobium parviflorum* | Onagraceae | Perennial | Native | Spring | P2 |
| *Oenothera biennis* | Onagraceae | Biennial | Alien | Spring & Autumn | P1 |
| *Ophioglossum vulgatum* | Ophioglossaceae | Perennial | Native | Spring & Autumn | P2 |
| *Spiranthes sinensis* | Orchidaceae | Perennial | Native | Spring | P1 |
| *Oxalis corniculata* | Oxalidaceae | Perennial | Native | Spring & Autumn | P1/P2/P3/P4 |
| *Oxalis corymbosa* | Oxalidaceae | Perennial | Native | Spring & Autumn | P1/P2/P3/P4 |
| *Phyllanthus urinaria* | Phyllanthaceae | Annual | Native | Spring & Autumn | P1/P2/P3/P4 |
| *Phytolacca acinosa* | Phytolaccaceae | Perennial | Native | Spring | P1 |
| *Phytolacca americana* | Phytolaccaceae | Perennial | Alien | Spring & Autumn | P1/P2/P3 |
| *Callitriche palustris* | Plantaginaceae | Annual | Native | Spring & Autumn | P1/P2 |
| *Linaria vulgaris* | Plantaginaceae | Perennial | Native | Spring | P2 |
| *Plantago asiatica* | Plantaginaceae | Perennial | Native | Spring & Autumn | P1/P2/P3 |
| *Plantago major* | Plantaginaceae | Perennial | Native | Spring | P1/P2/P3/P4 |
| *Plantago virginica* | Plantaginaceae | Annual | Alien | Spring & Autumn | P1 |
| *Veronica arvensis* | Plantaginaceae | Annual | Alien | Spring & Autumn | P1/P2/P3/P4 |
| *Veronica javanica* | Plantaginaceae | Annual | Native | Spring & Autumn | P1/P2/P3/P4 |
| *Veronica peregrina* | Plantaginaceae | Annual | Alien | Spring | P1/P2 |
| *Veronica persica* | Plantaginaceae | Annual | Alien | Spring & Autumn | P1/P2/P3 |
| *Corydalis decumbens* | Papaveraceae | Perennial | Native | Spring | P1/P2/P3 |
| *Alopecurus aequalis* | Poaceae | Annual | Native | Spring | P1/P2/P3 |
| *Avena sativa* | Poaceae | Annual | Native | Spring | P2 |
| *Axonopus compressus* | Poaceae | Perennial | Alien | Spring | P1 |
| *Cymbopogon citratus* | Poaceae | Perennial | Alien | Spring | P1 |
| *Cynodon dactylon* | Poaceae | Perennial | Native | Spring & Autumn | P1 |
| *Dactylis glomerata* | Poaceae | Perennial | Native | Spring | P2 |
| *Digitaria sanguinalis* | Poaceae | Annual | Native | Spring & Autumn | P1/P2/P3/P4 |
| *Eleusine indica* | Poaceae | Annual | Alien | Spring & Autumn | P1/P2 |
| *Imperata cylindrica* | Poaceae | Perennial | Native | Spring & Autumn | P2/P4 |
| *Poa annua* | Poaceae | Annual | Native | Spring & Autumn | P1/P2/P3/P4 |
| *Setaria viridis* | Poaceae | Annual | Native | Spring & Autumn | P1/P2/P3/P4 |
| *Pleuropterus multiflorus* | Polygonaceae | Perennial | Native | Spring | P1/P2 |
| *Polygonum aviculare* | Polygonaceae | Annual | Native | Spring & Autumn | P1 |
| *Rumex trisetifer* | Polygonaceae | Annual | Native | Spring & Autumn | P1/P2 |
| *Portulaca oleracea* | Portulacaceae | Annual | Native | Autumn | P1 |
| *Pteris multifida* | Pteridaceae | Perennial | Native | Spring & Autumn | P1/P2/P3 |
| *Clematis florida* | Ranunculaceae | Perennial | Native | Autumn | P2 |
| *Ranunculus sieboldii* | Ranunculaceae | Perennial | Native | Spring | P1/P2 |
| *Semiaquilegia adoxoides* | Ranunculaceae | Perennial | Native | Spring | P1/P2/P3/P4 |
| *Duchesnea indica* | Rosaceae | Perennial | Native | Spring & Autumn | P1/P2 |
| *Galium spurium* | Rubiaceae | Annual | Native | Spring | P1/P2 |
| *Hedyotis corymbosa* | Rubiaceae | Annual | Alien | Autumn | P1/P2 |
| *Paederia foetida* | Rubiaceae | Perennial | Native | Spring & Autumn | P1/P2/P3 |
| *Scleromitrion diffusum* | Rubiaceae | Annual | Native | Autumn | P1 |
| *Solanum americanum* | Solanaceae | Annual | Alien | Spring & Autumn | P1/P2/P3 |
| *Solanum nigrum* | Solanaceae | Annual | Native | Spring & Autumn | P1/P2/P3 |
| *Solanum undatum* | Solanaceae | Perennial | Alien | Spring | P1 |
| *Talinum paniculatum* | Talinaceae | Perennial | Alien | Spring & Autumn | P1/P2 |
| *Cyclosorus acuminatus* | Thelypteridaceae | Perennial | Native | Spring | P1 |
| *Cyclosorus parasiticus* | Thelypteridaceae | Perennial | Native | Spring & Autumn | P1/P3 |
| *Pilea peploides* | Urticaceae | Annual | Native | Spring | P1 |
| *Viola betonicifolia* | Violaceae | Perennial | Native | Spring | P2 |
| *Viola cornuta* | Violaceae | Annual | Alien | Spring | P2 |
| *Viola diffusa* | Violaceae | Annual | Native | Autumn | P1/P2 |
| *Viola inconspicua* | Violaceae | Perennial | Native | Autumn | P2 |
| *Viola philippica* | Violaceae | Perennial | Native | Spring & Autumn | P1/P2/P3/P4 |
| *Causonis japonica* | Vitaceae | Perennial | Native | Spring | P1 |

**Vegetation type^1^ :** plots were classified into four vegetation types based on the presence and combination of two dominant cultivated species, *Cynodon dactylon* and *Ophiopogon japonicus*: P1 (no cultivated species), P2 (*C. dactylon* only), P3 (*O. japonicus* only), and P4 (both species present).
